# The Economic Waste of Illness

**Published:** 1921-07-02

**Authors:** 


### THE PUBLIC WELL-BEING.

### The Economic Waste of Illness.

### FUTURE OF THE HEALTH MINISTRY.

"Illness," said the Minister of Health a few days ago, addressing members of the Pharmaceutical Society of Great Britain, "in addition to being a nuisance to the patient and an annoyance to his neighbours, is also a great economic waste to the community." That sentence, in brief, may be said to represent the new attitude of the public mind towards illness. The constant dinning of the elementary fact which it embodies into the minds of millions of people by a comparatively small number of people, by popular articles in the press, by the more frequent use of popular pamphlets, by the speeches of men whose knowledge and experience carry authority, even by the repetition of the truth in crisp newspaper head-lines, has really begun to take effect on the community at large, as well as on the average member of Parliament. The Ministry of Health has had an uphill fight. It has been variously described as an unwanted machine for interfering with the liberty of the individual, as an irritating instrument of State interference, as a dangerous dictatorship, as an expensive Departmental luxury created and maintained chiefly for the benefit of a new army of bureaucratic officials, as the supreme example of national incompetence and inefficiency. Up to quite recent times all these things and many more have been said of the new Ministry, and they have been said by politicians, publicists, and newspaper editors who ought to have known better.

But the Ministry of Health is beginning to come into its own. Of late there has been a distinct change in the tone of the general references to this great Department. The attacks have not ceased, but they have lost their virulence. Criticism continues, but it is better informed, and inspired more obviously with a desire to help rather than with the hope of destroying. There is a new note of sympathy with the laborious efforts of the Minister and his servants to establish a policy in health matters that is long-sighted and really national, to create a new public ideal in hygiene, to give new encouragement to preventive medicine in its wider aspects. The speeches in Parliament on both sides of the House of Commons, the tone of expert and popular articles in the press, all suggest something more than a dim glimmering of the intensely *practical* value of a healthy nation, of the intimate relation of health to industry, not only to the efficiency of industry, but to industry on its purely human side, and through that to the whole social fabric. A healthy nation is at once a prosperous and contented nation, and that is the essence of the matter in all the post-war effort initiated and carried through under immense difficulties by the Ministry which began its career under the guidance of that greatly maligned but faithful and far-sighted man, Dr. Addison. He and his Department have made mistakes, and the Ministry will undoubtedly make more mistakes—its scope is immense, and experiments must sometimes fail—but we are firmly convinced that it will develop into one of the very greatest permanent services of the State. The significant thing is that public recognition of the fact that health is economy is growing, and that it is no longer a popular "anti-waste stunt" to talk of public-health reform purely in the terms of pounds, shillings and pence expended, as if to spend money on making healthy mothers and healthy children, on attacking the causes of illness and disease, on producing hygienic conditions of work and everyday life, were as unproductive and as futile as building huge battleships at nine millions apiece.

The trend of recent thought was well suggested by Sir Leslie Mackenzie in his recent address to the Sanitary Science and Preventive Medicine Section of the Health Congress. The new objective in preventive medicine, he points out, is the production of fitness. That is one primary lesson of the war. If the signs of the time can be read even in part, they seem to indicate that in the coming century the races constituting the British people "will have to set themselves consciously and openly, under the guidance of the best science, not merely to master great infections and otherwise to prepare for themselves a healthy environment, but also to equip each individual person with strength to produce and courage to endure." If we are to maintain our pace in production we must be quite clear that the duties of civilised living, if not so apparently urgent as the duties of the war, are in the long run just as exacting and require for their performance the same high qualities of mind and body. There is no more illuminating study in the whole range of public health, said Sir Leslie Mackenzie, than the special investigation made during the war on industrial fatigue, bringing out just how the hours of labour, types of food, character of the work, and the general conditions of the factory show themselves in the form of increased or diminished output, as the case may be. The drift of the last thirty years has been from the study of environment to the study of individuals, and in medicine generally the drift has been from the study of the end products called disease to the incipient physiological deviations that need never become pathological. That significant observation led up to the expression of Sir Leslie Mackenzie's "dawning hope that in the years immediately before us the general public, by the improved organisation of the whole forces of research and treatment, will have the benefit of the highest medical skill informed by the latest medical science."

This great movement of progress will only be rendered possible, it may safely be added, by the whole-hearted co-operation of the State with every branch of the medical profession, and the full co-operation of the State, through the Ministry of

**THE PUBLIC WELL-BEING**—(*continued*).

Health, will in turn only be possible through the enlightened support of the whole community. That it will be difficult for the Ministry to avoid what are commonly called politics is proved by the point of view of men like Dr. C. C. Weeks, who regards "alcohol and alcoholism as one of the great coefficients in all the damaged life and disease met with day by day in the natural life of this country." Sir George Newman, Chief Medical Officer of Health, has said that if we are to have a healthy nation we must first deal with alcohol, then with venereal disease, and then with feeble-mindedness—but the three are hand-in-hand. The point is that immediately a Government Department like the Ministry of Health shows a realisation of the dangers of alcohol and proposes the adoption of a scientific method of combating the evil, it is bound to come up against vested interests and thus to create strong party opposition.

That is one of the many serious difficulties that will continue to beset a Department whose administrative work and constructive policy must necessarily touch the ordinary life of the people at numerous points. These difficulties will only be overcome by an informed public opinion, and by skilled and tactful, as well as bold, handling on the part of the Minister himself and those who serve him.

For a long time in these columns we have done our best to support the Ministry of Health in its widest intentions, while criticising its work in important details; at the same time, we have endeavoured to inform that special section of the public reached through our pages of the true relationship of the new Department of Health to the public and of its important bearing on the well-being of the community. We are glad to know from many persons whose opinion we respect that these efforts have been widely appreciated.

